# Sulfilimines: An Underexplored Bioisostere for Drug Design?

**DOI:** 10.1021/acs.jmedchem.5c00195

**Published:** 2025-01-31

**Authors:** Per I. Arvidsson

**Affiliations:** † Science for Life Laboratory, Drug Discovery & Development Platform & Division of Translational Medicine and Chemical Biology, Department of Medical Biochemistry and Biophysics, Karolinska Institutet, Stockholm 171 77, Sweden; ‡ Catalysis and Peptide Research Unit, University of KwaZulu Natal, Durban 4001, South Africa

## Abstract

Sulfilimines have so far received little attention as a bioisostere for sulfoxides in the design of biologically active compounds. A recent study on physicochemical and in vitro drug-like properties shows that sulfilimines deserve a place in the medicinal chemist’s toolbox.

In recent years, there has been a surge in research exploring novel synthetic routes to various sulfur derivatives,^1−4^ driven by the potential of these compounds to serve as innovative pharmacophores in drug design,^5,6^Figure 1A. This interest is perhaps best exemplified by an increasing number of clinical drug candidates that contain a sulfoximine functional group as an aza-analog of sulfones—a well-established functional group in many FDA approved drugs and marketed agrochemicals. In addition to sulfones and sulfonamides, the sulfoxide functional group is also well-represented in approved pharmaceuticals. In contrast, the pharmacology of aza-analogs of sulfoxides, i.e. a sulfilimine, is yet largely unexplored. In their Featured Article in this issue, Ellman and co-workers join forces with Gnamm to cast light on the potential of this novel functional group as a design element in pharmacologically active molecules.^7^ Their study meticulously examines essential drug-like properties such as solubility, metabolic stability, and membrane permeability, alongside the chemical and stereochemical integrity of sulfilimines. Furthermore, the incorporation of sulfilimine and related motifs into imatinib and a neutrophil elastase inhibitor provides a comparative analysis of properties in realistic drug-like molecules.

**Figure 1:**
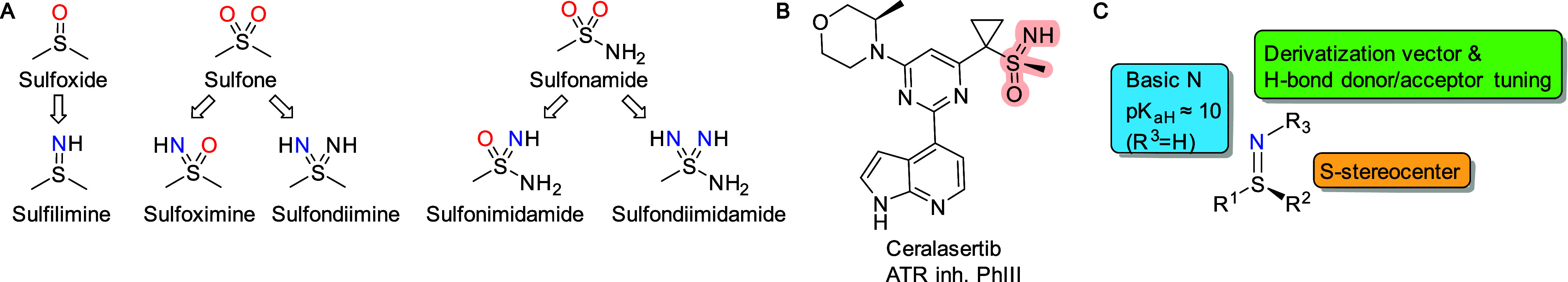
A) Representative bioisosteric replacements of sulfur-based sulfoxide, sulfone, and sulfonamide pharmacophores found in many FDA-approved therapeutics and crop-protection agents; B) ATR inhibitor Ceralasertib, now in PhIII clinical trials, is the most advanced sulfoximine containing human drug candidate; C) sulfilimines, studies by Ellman, Gnamm and co-workers in this issue, offer the medicinal chemist interesting opportunities for designing molecules with desired properties.

Bioisosteres are molecules or functional groups that have similar physical or chemical properties and produce broadly similar biological effects.^8^ In medicinal chemistry, bioisosteric replacements are important for creating new molecules with similar biological properties to a parent compound, while potentially improving various drug-like properties such as potency, selectivity, toxicity, or pharmacokinetics. Additionally, bioisostere replacement may offer ways to manage crowded intellectual property space. The exploration of novel bioisosteric functional groups is therefore crucial for the design and optimization of biologically active compounds. Sulfoximines have gained significant attention as bioisosteres in recent years, particularly as replacements for sulfones and sulfonamides, with Ceralasertib (Figure 1B), an ATR inhibitor in PhIII development by AstraZeneca, as a notable example. Similar to sulfoximines, but in contrast to sulfones, sulfilimines offer a stereogenic sulfur center, an additional vector for derivatization at the basic nitrogen atom that may be tuned as a hydrogen-bond donor or acceptor site per design, Figure 1C.

Reports on bioactive sulfilimines have so far been scarce, with only a handful of derivatives for crop protection, and even fewer for human targets, being reported. No systematic assessment of important physicochemical and in vitro drug-related properties therefore exists. By preparing a diverse set of smaller sulfilimine fragments, Gnamm, Ellman, and co-workers show that molecules containing a *S*-cyclopropyl or cyclobutyl substituent have high chemical stability, while *S*-alkyl, *S*-phenyl based sulfilimines generally have too poor chemical stability to be a preferred motif in medicinal chemistry. They also conclude that NH sulfilimines are configurational stable under physiological conditions, while the investigated *N*-acylated sulfilimine showed racemization at pH 1, but not at higher pHs. Sulfilimines with a free NH have similar basicity as tertiary amines with p*K*_aH_ ≈ 10. In terms of polarity, matched pair analysis shows that the order of polarity among bioisosteres generally follows: NH sulfilimines > sulfoximine > sulfoxide > sulfone; where *S*- and *N*-substituents may be used to tune polarity at will. The polar, low molecular weight fragments studied showed high metabolic stability in both mouse and human microsomes, as well as human hepatocytes—in addition, Caco-2 permeability was high with no observable efflux.

A more complex picture emerges when the preferred *S*-cyclopropyl sulfilimine functional group was incorporated into drug-like analogs of imatinib and a series of human neutrophil elastase (hNE) inhibitors, Figure 2.

**Figure 2:**
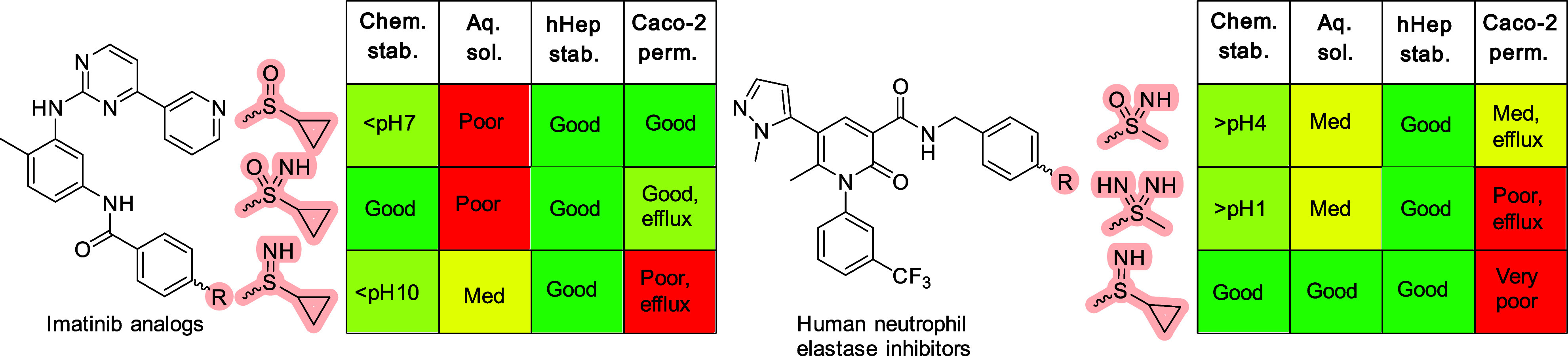
Representative data for selected imatinib- and human neutrophil elastase inhibitor-derivatives studies by Gnamm, Ellman, and co-workers.

Among the imatinib analogs, the higher polarity seen for the sulfilimine fragment vs the corresponding sulfoxide and sulfoximine, leads to an increase in aqueous solubility. The chemical stability of the sulfilimine is acceptable at physiological conditions, since only small amounts of degradation is seen at high pH. The stereogenic sulfur center was configurationally stable under the investigated conditions. Metabolic stability in human hepatocytes was high for all analogs shown in Figure 2 with the sulfilimine being the most stable among the three highlighted derivatives. The higher polarity of the unsubstituted sulfilimine vs the sulfoxide and sulfoximine also led to a lower degree of plasma protein binding (data not shown). The in vitro drug-like properties of the sulfilimine analog of human neutrophil elastase (hNE) inhibitors showed similar trends. In this case, the *S*-cyclopropyl sulfilimine was stable over the whole pH range studies, while the closest *S*-methyl sulfoximine and sulfondiimine analogs showed chemical instability at low pH values. Following the trend seen for imatinib analogs, sulfilimines with a free NH increased aqueous solubility and had high metabolic stability. As expected, the positive properties that follow low polarity also tend to compromise membrane permeability. This is unfortunately highly pronounced in the case of the sulfilimine analogs of both imatinib and human neutrophile elastase inhibitor. The imatinib analog with a free NH had low passive permeability through artificial PAMPA membranes, as well as low Caco-2 permeability with high active efflux. In the series of hNE inhibitors both the passive PAMPA permeability and Caco-2 cell permeability were too low to correctly quantify the active efflux that was also seen for the sulfoximine and sulfondiimine analogs.

The author’s viewpoint is that the methodical investigation of physicochemical and in vitro drug-like properties of sulfilimines by Gnamm, Ellman and co-workers has cleared the way for this underexplored functional group in the design of biologically active compounds, such as pharmaceuticals and agrochemicals. Especially *S*-cyclopropyl and cyclobutyl substituents proved to increase chemical stability, and in most cases, the stereogenic sulfur center was shown to be configurationally stable under physiological conditions. Sulfilimines with a free NH are characterized by desired drug-like properties such as high polarity, good solubility, high metabolic stability, and low plasma protein binding, but suffer from poor membrane permeability. Notably, low membrane permeability can be acceptable and even desired in medicinal chemistry design; low systemic exposure could be desired when locally targeting, e.g. the gastrointestinal tract, or when the route of administration is through inhalation or topical application. Another area where limited permeability could be used as a design element is around prodrugs with controlled release, e.g. liver targeting. The sulfilimine functionality also offers opportunities for functionalization of the nitrogen atom, which according to the data presented, dictates a lot of the properties for this functional group. For example, it would be interesting to explore if *S, N*-cyclized sulfilimines combine the positive properties seen here with higher membrane permeability. The opportunities for the sulfilimines are limited only by the ingenuity of the molecular architect!
